# Supplementary material for: A real-world demonstration of machine learning generalizability in the detection of intracranial hemorrhage on head computerized tomography
Source: Sci Rep. 2021 Aug 23;11:17051. doi: 10.1038/s41598-021-95533-2 (PMC8382750; doi:10.1038/s41598-021-95533-2)
Supplement: Supplementary file 1 — Supplementary Information. [file 41598_2021_95533_MOESM1_ESM.docx]

Supplementary Information

**Training and Validation Results**

Hyperparameters were set based on a grid-search. Below is a summary of the selected values for each model.

| **Model** | **Hyperparameters** |
| --- | --- |
| ResNext models | Batch size of 28, input image size of 512x512, Adam optimizer with a learning rate of 1.4e-4. |
| Catboost | Learning rate of 0.02, depth of 4, early stopping rounds of 100, training iterations of 20,000. |
| XGB | Learning rate of 0.08, depth of 5, log-loss evaluation metric. |
| LightGBM | 30 number of leaves, depth of 5, learning rate of 0.02, 5000 estimators, bagging frequency of 2 with a fraction of 0.7. |


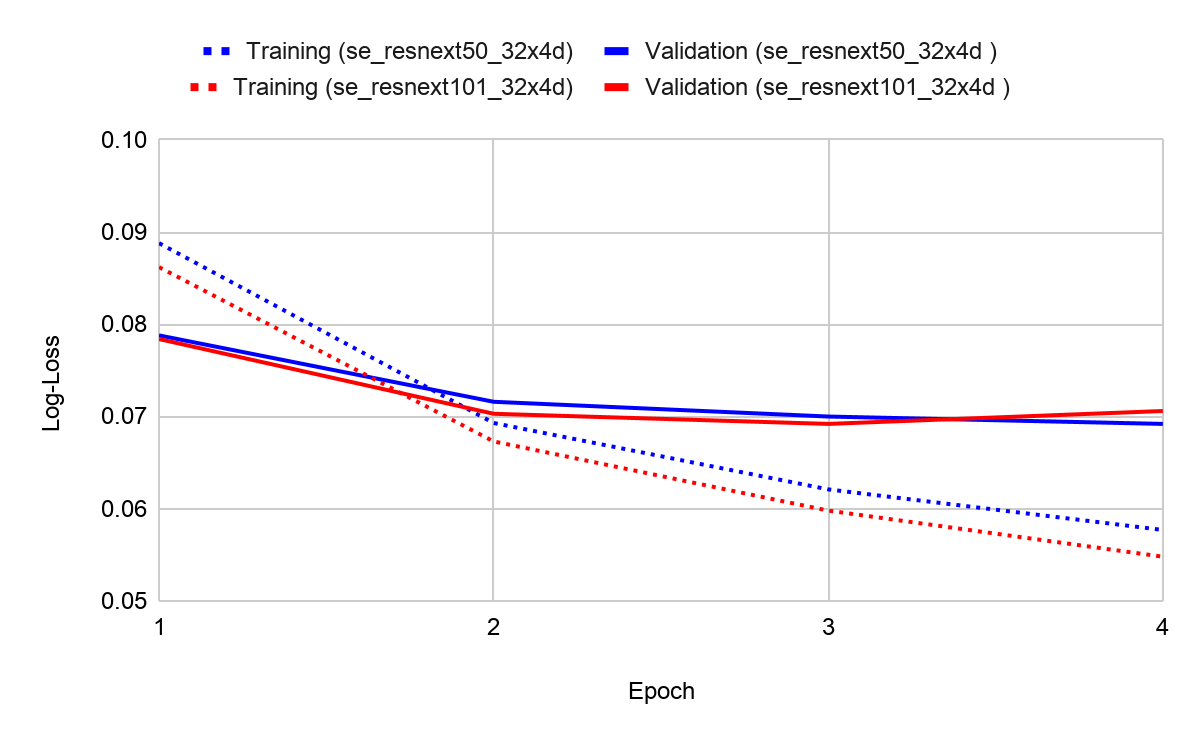


**Figure E1. Training and validation log-loss values of the ResNext models over 4 training epochs.**

**Table E1. Training log-loss values of the ResNext models for each hemorrhage type.**

| **Neural Network** | **EDH** | **SDH** | **SAH** | **IVH** | **IPH** | **Any** |
| --- | --- | --- | --- | --- | --- | --- |
| se_resnext50_32x4d | 0.0159 | 0.0856 | 0.0706 | 0.0281 | 0.0457 | 0.1075 |
| se_resnext101_32x4d | 0.0160 | 0.0855 | 0.0702 | 0.0272 | 0.0450 | 0.1075 |
| Ensemble | **0.0155** | **0.0838** | **0.069** | **0.0268** | **0.0441** | **0.1052** |

**Table E2. Training log-loss values of the boosting models for each hemorrhage type.**

| **Boosting Model** | **EDH** | **SDH** | **SAH** | **IVH** | **IPH** | **Any** |
| --- | --- | --- | --- | --- | --- | --- |
| LightGBM | 0.0149 | 0.0774 | 0.0619 | 0.0234 | 0.0387 | 0.0945 |
| Catboost | 0.0148 | 0.0779 | 0.0622 | 0.0236 | 0.0390 | 0.0948 |
| XGB | **0.0149** | **0.0775** | **0.0621** | **0.0235** | **0.0388** | **0.0948** |

**Figure E2. Calibration curve for hemorrhage classification tasks on the external dataset.**

**Table E3. ML performance in detecting ICH on the external validation set by gender (M: Male; F: Female). SEN: Sensitivity; SPEC: Specificity; PPV: Positive predictive value; NPV: Negative predictive value; AUC: Area under the receiver operating curve; Acc: Accuracy; BAcc: Balanced accuracy; MCC: Matthews correlation coefficient; F1: F1 score. All the values are in percent.**

| **Hemorrhage** | **SEN** | | **SPEC** | | **PPV** | | **NPV** | | **AUC** | | **Acc** | | **BAcc** | | **MCC** | | **F1** | |
| --- | --- | --- | --- | --- | --- | --- | --- | --- | --- | --- | --- | --- | --- | --- | --- | --- | --- | --- |
|  | **M** | **F** | **M** | **F** | **M** | **F** | **M** | **F** | **M** | **F** | **M** | **F** | **M** | **F** | **M** | **F** | **M** | **F** |
| **EDH** | 21.7 | 100 | 99.7 | 99.8 | 35.7 | 28.6 | 99.5 | 100 | 82.9 | 100 | 99.2 | 99.8 | 60.7 | 99.9 | 27.5 | 53.4 | 27.0 | 44.4 |
| **SDH** | 91.1 | 86.1 | 96.6 | 97.4 | 69.2 | 59.2 | 99.2 | 99.4 | 98.3 | 97.1 | 96.2 | 97.0 | 93.9 | 91.8 | 77.5 | 70.0 | 78.7 | 70.1 |
| **SAH** | 85.7 | 85.0 | 96.8 | 97.3 | 59.6 | 58.9 | 99.2 | 99.3 | 96.9 | 98.1 | 96.2 | 96.8 | 91.3 | 91.1 | 69.6 | 69.2 | 70.3 | 69.6 |
| **IVH** | 86.7 | 88.7 | 98.8 | 98.9 | 61.9 | 62.7 | 99.7 | 99.8 | 99.1 | 99.4 | 98.4 | 98.7 | 92.7 | 93.8 | 72.6 | 73.9 | 72.2 | 73.4 |
| **IPH** | 88.9 | 89.7 | 96.7 | 96.6 | 61.9 | 50.6 | 99.3 | 99.6 | 98.1 | 93.1 | 96.3 | 96.3 | 92.8 | 93.1 | 72.4 | 65.8 | 73.0 | 64.7 |
| **Any** | 90.1 | 92.0 | 93.3 | 95.1 | 66.9 | 65.1 | 98.6 | 99.2 | 96.4 | 97.0 | 93.0 | 94.8 | 92.1 | 93.5 | 74.2 | 74.8 | 77.1 | 76.2 |

**Table E4. ML performance in detecting ICH on the external validation set by age. SEN: Sensitivity; SPEC: Specificity; PPV: Positive predictive value; NPV: Negative predictive value; AUC: Area under the receiver operating curve; Acc: Accuracy; BAcc: Balanced accuracy; MCC: Matthews correlation coefficient; F1: F1 score. All the values are in percent.**

| **Hemorrhage** | **SEN** | | | | | **SPEC** | | | | | **PPV** | | | | | | **NPV** | | | | | **AUC** | | | | | **Acc** | | | | | **BAcc** | | | | | **MCC** | | | | | **F1** | | | | |
| --- | --- | --- | --- | --- | --- | --- | --- | --- | --- | --- | --- | --- | --- | --- | --- | --- | --- | --- | --- | --- | --- | --- | --- | --- | --- | --- | --- | --- | --- | --- | --- | --- | --- | --- | --- | --- | --- | --- | --- | --- | --- | --- | --- | --- | --- | --- |
|  | **Age < 20** | **20 ≤ Age < 40** | **40 ≤ Age < 60** | **60 ≤ Age < 80** | **Age ≥ 80** | **Age < 20** | **20 ≤ Age < 40** | **40 ≤ Age < 60** | **60 ≤ Age < 80** | **Age ≥ 80** | **Age < 20** | **20 ≤ Age < 40** | **40 ≤ Age < 60** | **60 ≤ Age < 80** | **Age ≥ 80** | **Age < 20** | | **20 ≤ Age < 40** | **40 ≤ Age < 60** | **60 ≤ Age < 80** | **Age ≥ 80** | **Age < 20** | **20 ≤ Age < 40** | **40 ≤ Age < 60** | **60 ≤ Age < 80** | **Age ≥ 80** | **Age < 20** | **20 ≤ Age < 40** | **40 ≤ Age < 60** | **60 ≤ Age < 80** | **Age ≥ 80** | **Age < 20** | **20 ≤ Age < 40** | **40 ≤ Age < 60** | **60 ≤ Age < 80** | **Age ≥ 80** | **Age < 20** | **20 ≤ Age < 40** | **40 ≤ Age < 60** | **60 ≤ Age < 80** | **Age ≥ 80** | **Age < 20** | **20 ≤ Age < 40** | **40 ≤ Age < 60** | **60 ≤ Age < 80** | **Age ≥ 80** |
| **EDH** | N/A | 38.5 | 0 | 25.0 | 25.0 | 98.7 | 99.7 | 99.7 | 99.8 | 99.9 | 0 | 55.6 | 0 | 20.0 | 50.0 | 100 | | 99.3 | 99.7 | 99.8 | 99.7 | N/A | 83.4 | 85.4 | 86.1 | 86.9 | 98.7 | 99.0 | 99.5 | 99.6 | 99.6 | 49.4 | 69.1 | 49.9 | 62.4 | 62.5 | N/A | 45.8 | 0 | 22.2 | 35.2 | 0 | 45.5 | 0 | 22.2 | 33.3 |
| **SDH** | 100 | 87.5 | 86.2 | 91.1 | 91.5 | 100 | 97.9 | 97.4 | 96.2 | 96.4 | 100 | 67.1 | 64.5 | 63.4 | 70.5 | 100 | | 99.4 | 99.2 | 99.3 | 99.2 | 100 | 97.8 | 97.2 | 98.2 | 98.4 | 100 | 97.5 | 96.8 | 95.8 | 96.0 | 100 | 92.8 | 91.8 | 93.7 | 94.0 | 100 | 75.4 | 73.0 | 74.0 | 78.3 | 100 | 76.0 | 73.8 | 74.8 | 79.6 |
| **SAH** | 75.0 | 81.3 | 94.6 | 91.7 | 67.2 | 94.7 | 98.0 | 96.7 | 96.8 | 96.9 | 42.9 | 67.6 | 58.8 | 59.4 | 52.9 | 98.6 | | 99.0 | 99.7 | 99.6 | 98.3 | 95.0 | 96.6 | 98.1 | 98.5 | 95.7 | 93.7 | 97.2 | 96.6 | 96.6 | 95.4 | 84.8 | 89.7 | 95.6 | 94.3 | 82.1 | 53.7 | 72.7 | 73.1 | 72.3 | 57.3 | 54.5 | 73.8 | 72.6 | 72.1 | 59.2 |
| **IVH** | 100 | 69.2 | 86.1 | 91.8 | 89.3 | 100 | 99.5 | 98.6 | 98.8 | 98.3 | 100 | 60.0 | 59.6 | 66.2 | 58.1 | 100 | | 99.7 | 99.7 | 99.8 | 99.7 | 100 | 99.6 | 98.7 | 99.5 | 99.4 | 100 | 99.2 | 98.3 | 98.6 | 98.1 | 100 | 84.4 | 92.4 | 95.3 | 93.8 | 100 | 64.0 | 70.9 | 77.3 | 71.2 | 100 | 64.3 | 70.5 | 76.9 | 70.4 |
| **IPH** | 66.7 | 88.9 | 89.9 | 90.0 | 89.5 | 97.3 | 97.9 | 96.3 | 96.2 | 96.7 | 66.7 | 61.5 | 56.3 | 55.6 | 59.3 | 97.3 | | 99.6 | 99.4 | 99.4 | 99.4 | 95.1 | 98.5 | 97.5 | 97.7 | 98.5 | 95.0 | 97.6 | 95.9 | 95.9 | 96.3 | 82.0 | 93.4 | 93.1 | 93.1 | 93.1 | 63.9 | 72.8 | 69.3 | 68.9 | 71.1 | 66.7 | 72.7 | 69.3 | 68.7 | 71.3 |
| **Any** | 83.3 | 88.1 | 89.3 | 93.9 | 91.5 | 95.6 | 96.3 | 93.1 | 93.7 | 93.5 | 62.5 | 67.9 | 61.2 | 67.5 | 69.3 | 98.6 | | 98.9 | 98.6 | 99.1 | 98.6 | 91.3 | 95.9 | 96.1 | 93.8 | 96.8 | 94.9 | 95.6 | 92.7 | 93.7 | 93.2 | 89.6 | 92.2 | 91.1 | 93.8 | 92.5 | 69.6 | 75.1 | 70.2 | 76.4 | 75.9 | 71.4 | 76.7 | 72.6 | 78.6 | 78.9 |
